# Definitive Chemoradiation Followed by Consolidation Immunotherapy for Unresectable Tracheal Squamous Cell Carcinoma: A Case Report

**DOI:** 10.7759/cureus.89737

**Published:** 2025-08-10

**Authors:** Adil Sayeed, Salman Hasan

**Affiliations:** 1 Radiation Oncology, Kansas College of Osteopathic Medicine, Wichita, USA; 2 Radiation Oncology, Ascension Via Christi St. Francis, Wichita, USA

**Keywords:** cancer immunotherapy, concurrent chemoradiation therapy, consolidation immunotherapy, durvalumab, pacific trial, radiation therapy, squamous cell carcinoma, tracheal cancer, unresectable cancer

## Abstract

Tracheal cancer is a rare malignancy that is typically treated with a multimodal approach with surgery, radiation therapy, and chemotherapy. Despite the treatment options, outcomes can be poor, with a high risk of recurrence that can be life-threatening. We present the case of a 68-year-old male with unresectable squamous cell carcinoma (SCC) of the trachea treated with definitive concurrent chemoradiation (66 Gy and cisplatin), followed by consolidation immunotherapy with durvalumab. This treatment approach mirrors the regimen used in unresectable stage III non-small cell lung cancer (NSCLC) based on the PACIFIC trial. The patient began durvalumab six weeks after chemoradiation and received five cycles over ~2.5 months, but therapy was discontinued early due to immune-mediated colitis presenting with diarrhea. The immunotherapy-related autoimmune side effects were successfully managed. He has since undergone routine surveillance with serial positron emission tomography/computed tomography (PET/CT) imaging and clinical follow-up and remains disease-free three years post-treatment. This case demonstrates the potential benefit of adapting an NSCLC immunotherapy regimen to primary tracheal squamous cell carcinoma (SCC), which has no standardized post-chemoradiation immunotherapy protocol.

## Introduction

Primary tracheal malignancies are rare neoplasms with an incidence of approximately 0.1 per 100,000 persons [1,2]. Primary tracheal malignancies have multiple subtypes, including squamous cell carcinomas and adenoid cystic carcinomas. Among these, squamous cell carcinoma (SCC) is the most common histologic subtype, typically affecting older men with a history of smoking [3]. Squamous cell tracheal malignancies clinically present with hemoptysis, dysphagia, and hoarseness, but symptoms are largely dependent on the location of the tumor [4,5].

Since the clinical symptoms are nonspecific, it may be mistaken for conditions like asthma, chronic obstructive pulmonary disease (COPD), and pneumonia [2,4]. Furthermore, about 5-10% of cases may be asymptomatic [6]. Due to non-specific symptoms, tracheal cancers are often detected at an advanced stage, leading to a poorer prognosis. It has been reported that on average, the duration of delay is 12 months [7]. Depending on the degree and characteristics of the tumor, it could be treated with surgical resection, radiation, or chemotherapy. Surgical resection is viewed as the primary curative treatment method for tracheal tumors [2,8]. However, resection may not be feasible in certain conditions, such as when the tumor is too large or its removal would compromise the structural integrity of the trachea. It has been reported that the mean survival time for resected SCC was 38 months, compared to 8.8 months with unresected cases; the five-year overall survival for resected tracheal SCC was reported to be 39.1%, and only 7.3% for unresectable cases treated non-surgically [9].

Immunotherapy has become a cornerstone in the treatment of many malignancies, improving outcomes such as survival and reducing recurrence risk. In NSCLC, consolidation immunotherapy with durvalumab following definitive chemoradiation significantly improved survival in the PACIFIC trial and is now the standard of care for unresectable stage III NSCLC. In contrast, tracheal cancers lack a standardized post-chemoradiation immunotherapy protocol [10]. Given the rarity, aggressive nature, and poor prognosis of tracheal cancers and the absence of clinical trials, only a few reports have documented the use of immune checkpoint inhibitors in this setting. Here, we present a case of unresectable primary tracheal SCC treated with concurrent chemoradiation followed by consolidation durvalumab, highlighting a potential application of the PACIFIC trial to this rare disease.

## Case presentation

A 68-year-old male with a past medical history of COPD, hyperlipidemia, hypertension, and a 0.5 pack/day smoking history presented to his physician with several months of cough, dyspnea, and wheezing. He had a significant family history of a mother who was diagnosed with pancreatic cancer. He had a heart rate of 57 beats per minute and a blood pressure of 113/53 mmHg. In addition, the respiratory rate was 18/min, peripheral oxygen saturation was 96%, and temperature was 97.6°F. The symptoms were initially attributed to worsening COPD, but the lack of improvement despite treatment prompted further imaging.

A chest computed tomography (CT) approximately 3-4 months from symptom onset indicated the presence of a 1.6 x 1.6 lobulated focus on the trachea at the level of the carina. A bronchoscopy was performed, and a tracheal mass was found.

**Figure 1:**
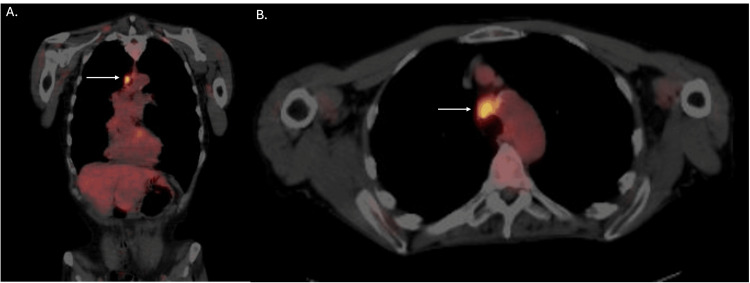
Pre-treatment FDG-18 PET/CT of the chest. (A) Coronal view. (B) Axial view. Imaging reveals a 3.25 x 2.5 cm hypermetabolic mass in the anterior trachea with an SUVmax of 7.65

Laboratory examinations of the mass were conducted, and a positron emission tomography (PET)/CT with FDG-18 was performed to assess the extent of the malignancy. PET/CT findings indicated an approximately 3.5 x 2.5 cm partially calcified hypermetabolic mass at the low anterior trachea, demonstrating increased metabolic activity, consistent with a primary neoplasm (Figure 1). The maximum standardized uptake value (SUVmax) was found to be 7.65, with slight nodular uptake about the right hilum with an SUVmax of 3.63. The very slight asymmetric increased nodular uptake in the right hilum is indeterminate but concerning hypermetabolic lymphadenopathy. Imaging of the soft tissues of the neck, visualized brain, and osseous uptake were negative, indicating no metastasis at those locations.

There was abnormal soft tissue density occupying a large portion of the right lower lobe bronchus, and a somewhat patchy nodular infiltrate within the right lower lobe was noted. However, since these areas do not demonstrate hypermetabolism, it does not indicate neoplasms, but it is likely that they reflect secretions and post-obstructive pneumonia.

A second bronchoscopy was performed to obtain a biopsy and to perform cryotherapy to restore patency in the trachea. The bronchoscopy noted the presence of a tumor in the mid- to lower trachea occupying about half the cross-sectional area. Histopathologic examination of the biopsy of the tumor confirmed invasive, well-to-moderately differentiated G1-G2 keratinizing squamous cell carcinoma.

**Figure 2:**
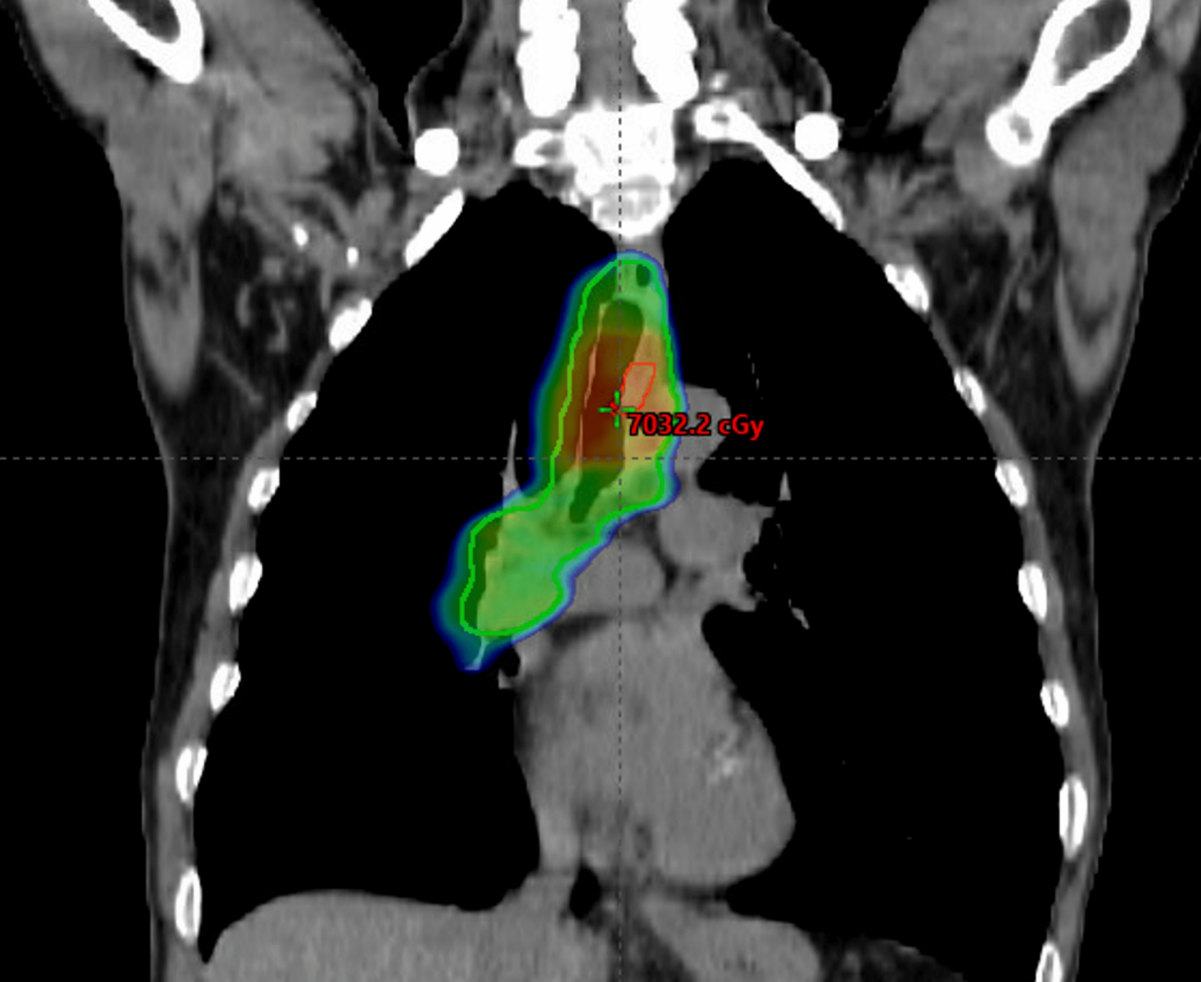
Coronal radiation dose distribution for radiation therapy. The green area indicates the planning target volume of 60 Gy, and the red area indicates the gross tumor volume of 66 Gy

After discussions about his candidacy for surgical resection, it was determined that he was not a candidate for surgical resection due to the proximity to the carina and the length of trachea involved that would require an extensive amount of tracheal reconstruction. He was recommended definitive concurrent chemoradiation with 33 fractions of 66 Gy (given five times per week) and weekly cisplatin. The radiation dose prescription was 60 Gy in 30 fractions to the clinical target volume that included the at-risk areas of the trachea, the gross tumor volume, and part of the right hilum, since there was a slight asymmetric increased nodular uptake. An additional 6 Gy in three fractions was prescribed to the GTV to a total dose of 66 Gy in 33 fractions (Figure 2). He tolerated chemoradiation well and did not experience any significant symptoms.

**Figure 3:**
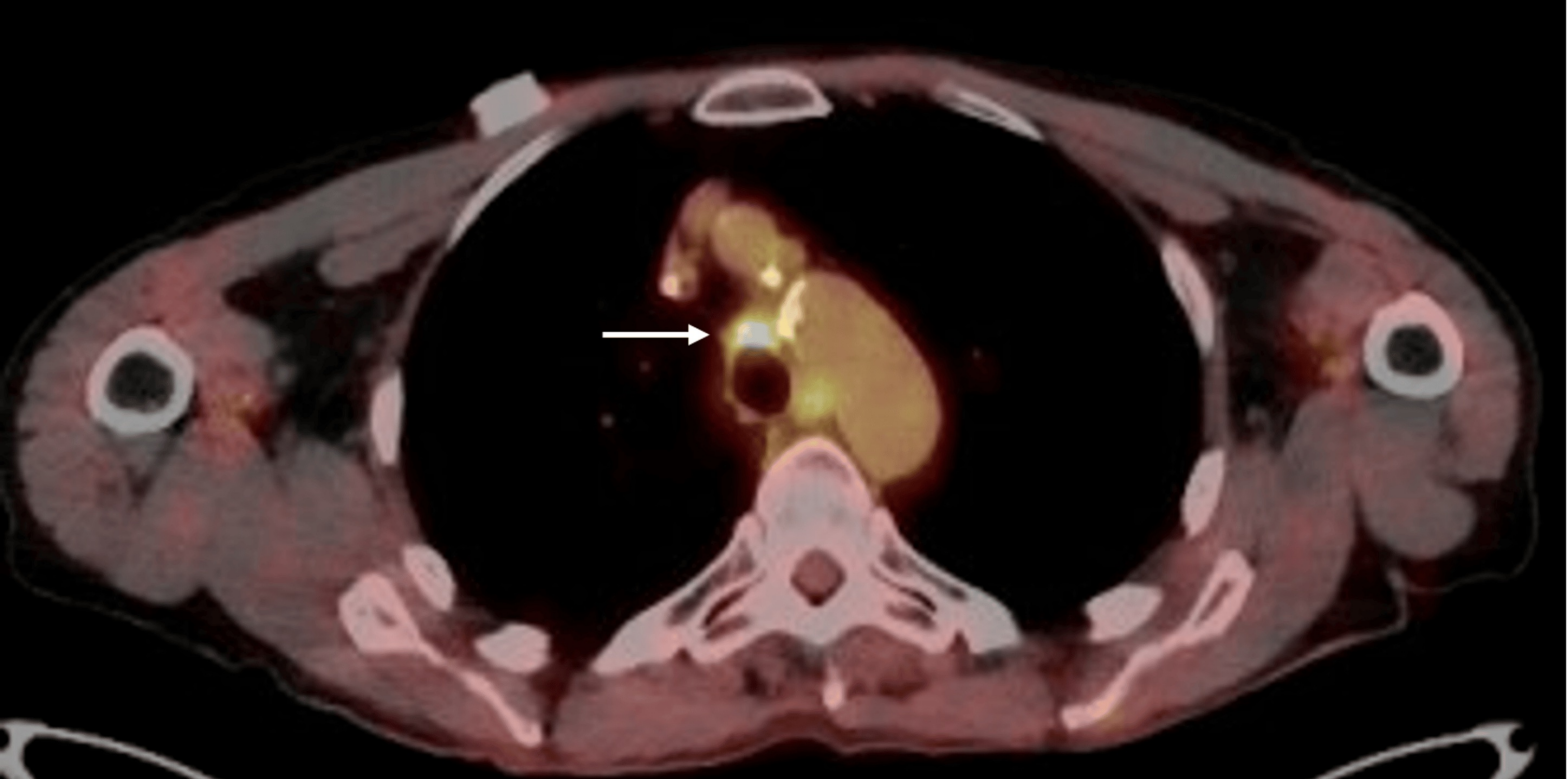
Axial PET/CT of the chest with FDG-18 3 months post-chemoradiation shows a decrease in size of the hypermetabolic mass with an SUVmax of 4.5

Six weeks after chemoradiation, our patient was started on five cycles of consolidation immunotherapy with durvalumab to combat the high rate of recurrence. A PET/CT three months post-chemoradiation, and during the immunotherapy treatment, revealed a decrease in the SUVmax to 4.5 (Figure 3). There was an adverse autoimmune side effect of uncontrolled diarrhea three months into treatment that the patient could not tolerate and required the discontinuation of the immunotherapy. Initial treatment with loperamide failed to control the diarrhea, and subsequent corticosteroid therapy with prednisone and budesonide was also ineffective; ultimately, symptoms resolved following administration of infliximab.

**Figure 4:**
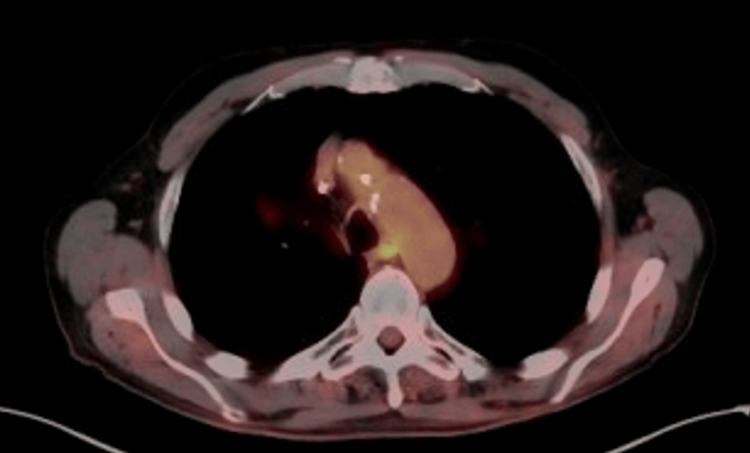
Axial PET/CT of the chest with FDG-18 20 months post-chemoradiation and 15 months post-immunotherapy does not indicate the presence of a hypermetabolic mass

Our patient has been undergoing surveillance imaging with CT and PET/CT scans approximately every 3-4 months to closely monitor and detect for signs of recurrence, and thus far, there have been none. The most recent PET/CT, performed 20 months post-chemoradiation and 15 months post-immunotherapy, did not demonstrate recurrent disease (Figure 4). He reports near-normal respiratory function and has returned to his usual daily activities. A summary of SUVmax values and corresponding imaging findings at each time point is presented in Table 1.

**Table 1:** Serial PET/CT SUVmax measurements and imaging findings throughout treatment and follow-up

Time Point	Clinical Context	SUVmax	Imaging Findings
Baseline (Pre-treatment)	Prior to chemoradiation	7.65	3.25 × 2.5 cm partially calcified hypermetabolic tracheal mass
3 months post-chemoradiation (during immunotherapy)	On durvalumab	4.5	Decrease in size of hypermetabolic tracheal mass
20 months post-chemoradiation / 15 months post-immunotherapy	Surveillance imaging	Not detectable	No hypermetabolic mass or evidence of recurrence

## Discussion

Tracheal squamous cell carcinomas are rare cancers that are challenging to treat due to their low incidence and high risk of recurrence despite optimal treatment. Recognizing tracheal cancer is also difficult with regard to its nonspecific symptoms. The diagnostic criteria depend on histopathologic examination, and the management of tracheal SCC is determined by the extent of disease spread. Furthermore, given the rarity of tracheal SCC, there is a lack of randomized controlled trials to establish the most effective therapy for this condition [11,12]. Complete surgical resection with negative margins offers the best chances for cure [9]. However, cases may be unresectable due to tumor extent, location, or comorbidity. Our patient's tumor exemplified this, as it was unresectable due to the location at the carina and the extensive length of trachea that would require complex reconstruction.

For tracheal tumors where surgery is feasible, a five-year survival rate of 39% is reported. The 5- and 10-year survival rates for unresectable SCC of the trachea are 7% and 5%, respectively [9]. These cancers have the potential to spread to other tissues, and worse outcomes are associated with regional and distant metastases [6,9]. Our patient had an SCC that remained locally aggressive and showed no signs of metastasis.

The purpose of immunotherapy in tracheal SCC is to combat the high rate of recurrence. There have been successful reports demonstrating the use of immunotherapy-based treatment for the treatment of tracheal SCC with nivolumab and pembrolizumab [13,14]. The PACIFIC trial demonstrated the role of immunotherapy with durvalumab, a PDL-1 inhibitor, following platinum-based chemoradiation for NSCLC with decreased risk of recurrence and improved overall survival [10]. Our patient’s case illustrates that consolidation immunotherapy with durvalumab following concurrent chemoradiation could be an effective therapeutic option for the treatment of tracheal SCC.

Furthermore, this patient's experience highlights important toxicity considerations. Consolidation durvalumab is generally well tolerated; however, immune-related adverse effects, though uncommon, can occur. Diarrhea-like symptoms were rarely reported with durvalumab in the PACIFIC case, affecting about 1.1% of patients [15].

## Conclusions

Unresectable tracheal squamous cell carcinoma is a rare and aggressive malignancy with historically poor outcomes and may be challenging to treat. Current treatment options include surgery, radiation therapy, and chemotherapy. This case demonstrates the potential benefit of adapting a proven NSCLC treatment approach (definitive chemoradiation followed by consolidation immunotherapy with durvalumab) to tracheal SCC. This patient achieved a sustained complete remission, suggesting that durvalumab can reduce recurrence risk in this setting. While generally well tolerated, durvalumab carries a risk of immune-related adverse events. The risk of serious immune-related toxicity (such as colitis in our patient) must be recognized and managed with appropriate interventions.

To our knowledge, there are no prior published reports of concurrent chemoradiation followed by consolidation durvalumab for primary tracheal SCC. A few isolated case reports describe the use of immune checkpoint inhibitors such as pembrolizumab or nivolumab in recurrent or metastatic tracheal SCC; however, none have combined this with definitive chemoradiation or used durvalumab in the consolidation setting. Thus, our case adds to the limited literature and suggests further investigation into extrapolating PACIFIC-based immunotherapy strategies to rare, unresectable tracheal tumors, with prospective studies warranted to evaluate their generalizability.
